# Effect of gamma-mangostin on testosterone levels in Leydig cell culture of Sprague-Dawley rat induced by advanced glycation end products: a preliminary study

**DOI:** 10.1186/s12919-019-0173-x

**Published:** 2019-12-16

**Authors:** Dicky Moch Rizal, Aditya Rifqi Fauzi, Rustamaji

**Affiliations:** 1 Department of Physiology, Faculty of Medicine, Public Health and Nursing Universitas Gadjah Mada/Dr. Sardjito Hospital, Yogyakarta, 55281 Indonesia; 2 Faculty of Medicine, Public Health and Nursing, Universitas Gadjah Mada/Dr. Sardjito Hospital, Yogyakarta, 55281 Indonesia; 3 Department of Pharmacology and Therapy, Faculty of Medicine, Public Health and Nursing Universitas Gadjah Mada, /Dr. Sardjito Hospital, Yogyakarta, 55281 Indonesia

**Keywords:** Testosterone level, Leydig cell culture, Sprague-Dawley rat, Advanced glycation end products, Gamma-mangostin

## Abstract

**Background:**

Advanced glycation end products (AGE) is a toxic compound in the human body that can deteriorate health and induce an inflammatory response. One of the type of cells affected is Leydig cells, cells that produce testosterone and located in interstitial areas of the testes. Pericarp extract of *Garcinia mangostana* contains an antioxidant compound called gamma-mangostin that can decrease inflammatory responses and toxic effects of AGE. We aimed to compare the testosterone levels in Leydig cell culture of Sprague-Dawley rat induced by AGE only and following gamma-mangostin.

**Methods:**

An experimental laboratory study was conducted on testosterone level in Leydig cell culture of Sprague-Dawley rats induced by advanced glycation end products 200 μg/mL and given gamma-mangostin 5 μM compared to cell cultures that were not given gamma-mangostin.

**Results:**

Nine Leydig cell cultures were ascertained and divided into three groups. No significant difference was found in the testosterone level of Leydig cell culture given AGE only (1.33 ng/10^5^ cells/24 h) compared to the group given AGE and gamma-mangostin (1.30 ng/10^5^ cells/24 h) (*p* = 0.535).

**Conclusion:**

The testosterone level in Leydig cell cultures induced by AGE were lower than those not given, but similar in the AGE-only group and group given gamma-mangostin. The mean testosterone levels in all groups were in the range of expected levels (0.025–15 ng/10^5^ cells/24 h). Further study with larger samples is important to clarify and confirm our findings.

## Background

The hormone testosterone is an essential part of men’s health. Testosterone has a major function in the process of spermatogenesis and the formation of male secondary sexual characteristics, but many other functions are equally important, such as helping increase bone and muscle mass, inhibiting ageing, preventing cardiac arrhythmias, increasing fat metabolism, and preventing atherosclerosis. Testosterone is produced by Leydig cells, which are cells located in the interstitial testes, between seminiferous tubules. However, recently there has been an increase in the prevalence of hypogonadism in the elderly population, which occurs in as much as 20% in men aged 60–70 years and 50% at the age of more than 80 years [1, 2]. The World Health Organization (WHO) states that one in four couples in developing countries experience infertility and sexual dysfunction [3].

Several studies show that male reproductive health problems are the most common cause of couples with infertility cases. In Indonesia, there are 12% or about 3 million infertile couples. It is known that 30% of all cases of infertile couples are caused by men and tend to increase with older couples to 40% [4].

Infertility in men can be caused by a variety of factors, including infection, tumors, hormonal imbalances, smoking and obesity [5]. The most common cause is oxidative stress caused by an increase in Reactive Oxygen Species (ROS) in the testes and a decrease in antioxidant agents that disrupt the process of spermatogenesis [6]. Oxidative stress can significantly interfere with sperm function, which is an early sign of infertility in men [7].

One of the compounds that can cause the formation of ROS is Advanced Glycation End products (AGE). AGE is a toxic compound derived from proteins or lipids that undergo the process of glycation after binding to sugars. AGE can trigger damage to blood vessel walls, cardiovascular disease, neurodegenerative disorders, cancer and nonalcoholic steatohepatitis through inflammatory mechanisms [8, 9].

Mangosteen, which has the Latin name *Garcinia mangostana*, has long been used as a medicine to treat skin infections, wounds and diarrhea in Southeast Asia because it contains anti-inflammatory compounds. In one study, it was found that the mangosteen pericarp contained gamma-mangostin, a derivative of xanthones which can reduce the inflammatory reaction by reducing the expenditure of prostaglandin E2 [10]. The use of mangosteen pericarp extract containing gamma-mangostin is already familiar in Indonesia because it is known to have many health benefits. Beginning with increasing cases of infertility caused by a decrease in testosterone hormone, the authors are interested in examining testosterone levels in Leydig cell cultures of Sprague-Dawley rats induced by AGE 200 μg/mL and given gamma-mangostin 5 μM.

## Methods

### Samples

This study was conducted from April 2014–May 2015 at Cell culture laboratory, Department of Physiology, Faculty of Medicine, Public Health and Nursing, Universitas Gadjah Mada, Yogyakarta, Indonesia. An in vitro experimental laboratory study was conducted with samples of male Sprague-Dawley rats aged 90 days, weighing around 300–350 g.

This study was approved by the Institutional Review Board of the Faculty of Medicine, Public Health and Nursing, Universitas Gadjah Mada, Yogyakarta, Indonesia (KE/FK/342/EC/2015).

#### Testis retrieval

**Fig. 1:**
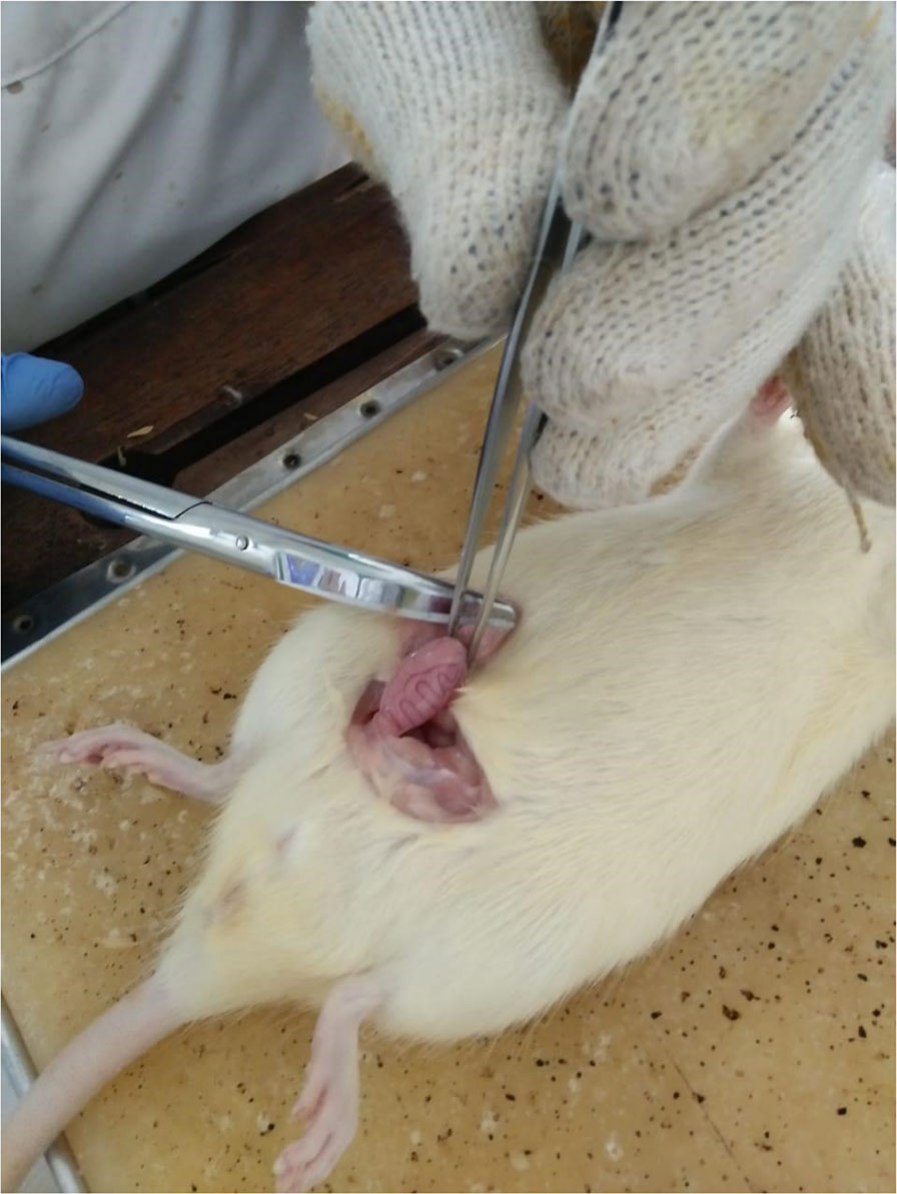
Testes retrival from Sprague-Dawley rat

We used animal handling guidelines, the common surgical procedures in rodents from Foley [11]. The rats were fasted for about 10 h before removing the testicles. Then, the rats were anaesthetized using ketamine HCl 0.3 mL/100grBW intramuscularly. After being unconscious, the four limbs of the rat were fixed using a rope on the operating table. Hairs in the abdomen and testicles were moistened using wet cotton and then shaved until the skin appears as limited as the area to be opened. The area to be opened was sterilized with an alcohol swab and then incised about 2 cm along the midline of the abdomen with a scalpel. A peritoneal incision of 1.5–2 cm long was made. Using a pair of curved tweezers and small scissors, a skin incision was made midline along the lower abdomen about 0.5 in. anterior to the genitals, and about 1.0 cm long. The skin was opened towards the right and left to remove each of the testicles from just one incision. Two vas deferens were then identified on the side of the testis. The left vas deferens was gently grasped with the forceps and then partially lifted so that the incision was clearly visible (Fig. 1).

The vas deferens was then ligated and cut as described above. After the testis was taken, the wound area was cleaned and observed for any bleeding. The peritoneum and the skin were re-sewn with absorbable threads. Next, the stitches were smeared with povidone-iodine and allowed to dry. Finally, the rats were euthanized by administering lethal dose of ketamin 0.45 mg/kg.

#### Making AGE-BSA preparations

AGE-BSA is AGE derived from Bovine Serum Albumin (BSA), which is reacted with glucose and incubation for several weeks. The AGE-BSA used in this study is the initial preparation of powder, which was then dissolved using phosphate-buffered saline (PBS). The AGE-BSA used in this study is a Biovision product with catalogue number 2221–10 and has a specificity of 98%. This product shows an AGE content of 7000% compared to ordinary BSA.

#### Leydig cell isolation and culture

In this procedure, we used guidelines from the previous study by Sun et al. [12]. Epididymis, visible blood vessels, fat and other connective tissue are removed carefully from the testes with microscissors. The tunica albuginea was then dissected out, and a pair of testicles from each rat were incubated into a 50 ml centrifuge tube. Testes were then placed in a mixture of previously cooled Dulbecco’s modified Eagle medium (DMEM)/Ham’s F12 Media (Sigma-Aldrich, Merck KGaA, Darmstadt, Germany) 1:1, then they were mixed with 15 mM NaHCO3, 20 mM HEPES, pH 7.4; 100 U/ml penicillin, 2.5 pg/ml amphotericin B and 0.1% BSA, with temperature continuously maintained in the laboratory with ice. All solutions were sterile, and all procedures done in sterile conditions.

Testicular tissue pieces were isolated for the process of isolating Leydig cells and placed in fresh medium, and then tunica albuginea was removed. The tissue was then rinsed three times with the media and finely chopped in a petri dish.

Tissue fragments were placed in a solution of 0.04% collagenase (type I, Sigma Chemical Co., 130 U/mg) and 1.0 μg/ml trypsin inhibitors in the culture media mentioned above, under constant agitation at 34 °C for 40 min.

After this procedure, the collagenase solution was diluted four times with a culture medium, and a small piece of tissue was inserted for sedimentation for 10 min. The supernatant was centrifuged at room temperature for 3 min at 200 g, and the cell pellets were washed twice and then stored in fresh tissue culture media. Administration of both suspension for 30 min with the same conditions was done on the remaining tissue pieces. Cells were collected and washed as described in the procedure above.

**Fig. 2:**
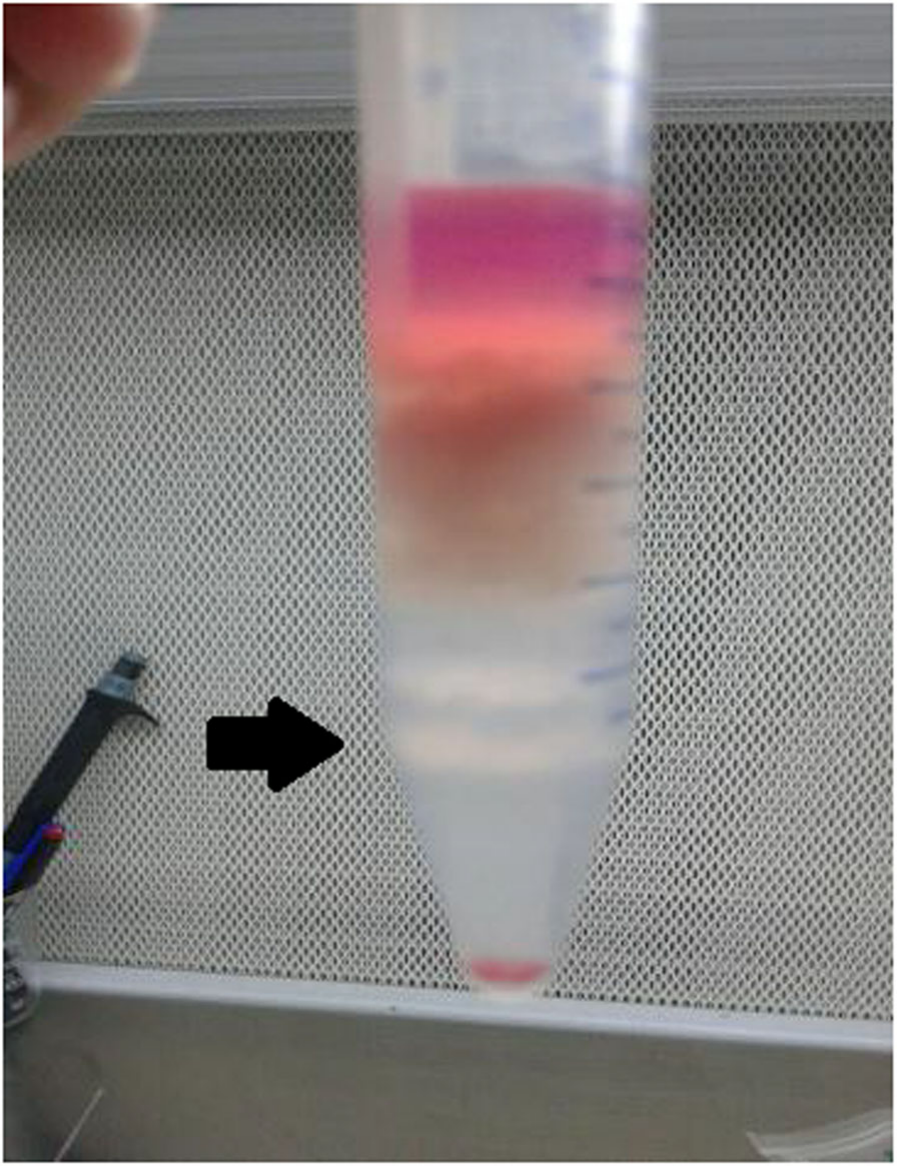
After the final centrifugation process, Leydig cells will form a ring-like layer between 40 and 60% gradient medium concentration. The black arrow indicates the Leydig cell-rich layer

The suspension obtained from the two collagenase treatments was combined, and the sedimentation produced from the 10 and 30-min treatments were taken to eliminate the remaining tubular segments. Initial cells were obtained from the supernatant from the use of 4-layer percoll gradients (21, 26, 34 and 60%). The gradient was centrifuged at 800 g for 30 min at room temperature. The layers formed between 40 and 60% concentrations were taken (Fig. 2) and washed with a medium to remove the Percoll medium. Viability was confirmed by a separate, more than 90% Tryptan Blue test.

**Fig. 3:**
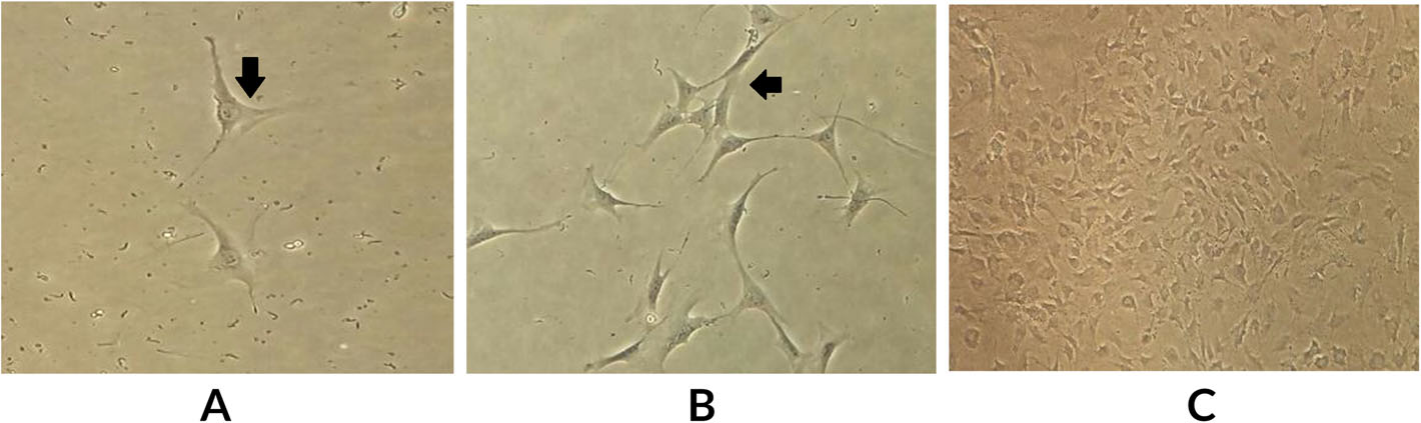
Leydig cell culture at days 2, 4 and 7. Black arrows indicate Leydig cells. **a**. The appearance of Leydig cells in culture 24–48 h: Leydig cells are still less. Leydig cells that have grown in polygonal form, **b**. The appearance of Leydig cells in culture in 96 h. Leydig cells are increasing in number. **c** Leydig cell appearance in 156 h. Leydig cells have proliferated rapidly and reached confluent of about 80%. Culture was observed using an inverted microscope at 100x magnification

Approximately 2 × 10^5^/cm^2^ cells were planted in the well in the DMEM/Ham’s F12 media, and then were added 15 mM NaHCO_3_, 20 mM HEPES, pH 7.4; 100 U/mI penicillin, 2.5 pg/ml amphotericin B, 10 μg/ml transferrin; 5μg/ml hydrocortisone and 2% Fetal Bovine Serum (Sigma-Aldrich, Merck KGaA, Darmstadt, Germany). Culture is conducted in an incubator which was regulated at 34 °C with a pressure of 5% CO_2_ in the air. Cells were estimated to coagulate in the media 24 h after culture. In the initial period, the cells will stick together in the first hours of culture and begin to grow, then began to close to confluence in 7 days (Fig. 3).

#### Testosterone levels measurement

Testosterone concentration in the media was examined by ELISA according to the procedure. The fluid to be examined was dripped with 20 μL of standard fluid (biotinylated polyclonal antibody) and then drops of 200 μL of the conjugate enzyme were added and 100 μL of the testosterone derivative labelled with the ruthenium complex. The microparticles in the mixture were captured by electrodes, which then induced chemiluminescent emissions, and the results were calculated with the photomultiplier recorded in ng/10^5^ cells/24 h. The measurement validity was 0.025–15 ng/10^5^ cells/24 h (Roche).

#### Preparation of gamma-mangostin

Gamma-mangostin is a xanthone derivative derived from mangosteen pericarp extract (*Garcinia mangostana*). Gamma mangostin used in this study is a product from Sigma-Aldrich with MG 6824 catalogue numbers in powder preparations which are then dissolved with dimethyl-sulfoxide (DMSO) to obtain a concentration of 20 mM as a solution and then diluted as needed. Gamma-mangostin has a purity level of 98%.

### Statistical analysis

All data were recorded in a computer database and analyzed using the SPSS Statistics 22 program (SPSS Inc., Chicago, IL, USA). The results are displayed as mean values ± standard deviation (SD). Mean differences between groups were analyzed using one-way ANOVA test. Post-hoc analysis was performed using the LSD method to determine the groups that had mean differences. A *p-*value < 0.05 was considered statistically significant.

## Results

### Association between gamma-mangostin, AGE, and testosterone level

Table 1 shows that the average testosterone level is higher in group 2 (Leydig cell + AGE-BSA cell culture 200 μg/mL) than group 3 (Leydig cell + AGE-BSA cell culture 200 μg/mL + gamma-mangostin 5 μM) and the highest at group 1 (control) with a significant difference (*p* = 0.036).

**Table 1:** Testosrerone level mean values

Variable	Group 1 Control (*n* = 3)	Group 2 AGE-BSA 200 μg/mL (*n* = 3)	Group 3 AGE-BSA 200 μg/mL + gamma-mangostin 5 μM (*n* = 3)	*p-*value
*Testosterone levels* (ng/10^5^ cells/24 h)	1.47 ± 0.05	1.33 ± 0.03	1.30 ± 0.10	0.036*

Data were served in mean ± SD

*, *p* < 0.05 is considered statistically significant. *AGE-BSA* Advanced glycation end products-Bovine Serum Albumin

After a post-hoc analysis with the LSD test, significant differences (*p* < 0.05) were found in the comparison of the group 1 (control) group with group 2 (AGE-BSA 200 μg/mL group) and group 1 (control) with the group 3 (AGE-BSA group 200 μg/mL + gamma-mangostin 5 μM).

**Fig. 4:**
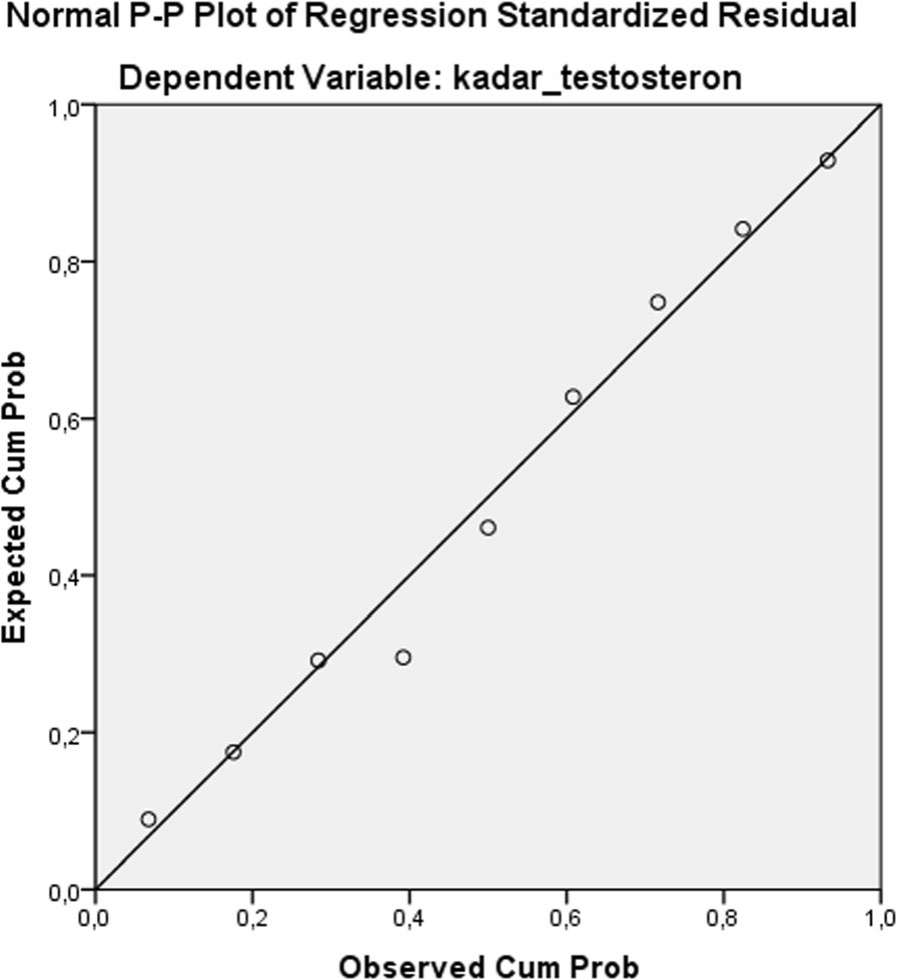
Linearity graphic of testosterone level

Linearity test was done on testosterone level data, showing a significance value of 0.297, which means there is a significant linear relationship between the dependent variables (testosterone levels) and independent variables (AGE-BSA and gamma-mangostin). Linearity graphic shown in Fig. 4.

## Discussion

Based on the results of the research conducted, induction of AGE 200 μg/mL in Leydig cell culture in Sprague-Dawley rat showed that the lowest average testosterone level was in group 3 (1.30 ng/10^5^ cells/24 h) and the highest in group 1 (1.47 ng/10^5^ cells/24 h) with a significant difference (*p* = 0.036). This significant difference means that intergroup testosterone levels are relatively different. Based on post-hoc analysis with LSD test, the results showed that the main differences were between group 1 and group 2 (*p* = 0.039) and group 1 with group 3 (*p* = 0.016).

From the results obtained, it can be seen that the decrease in testosterone levels in group 3 is not significant when compared to group 2. This finding shows that gamma-mangostin can inhibit the oxidation process caused by AGE according to the theoretical basis so that Leydig cell cultures can still produce testosterone accordingly at an expected level. In a study conducted by Jung [12], it was found that the mangostin compound had an antioxidant effect on rat breast preneoplastic lesions at IC50 2.44 μM, while in this study gamma-mangostin levels were used at 5 μM.

Murugesan [13] conducted a study of Leydig cell culture induced by polychlorinated biphenyl (PCB) and was given vitamins C and E. In this study it was found that there was a decrease in testosterone production in vitro due to a decrease in steroidogenic enzyme activity and the number of luteinizing hormone (LH) receptors caused by PCBs in conditions basal and LH stimulated. There was also an increase in ROS and lipid peroxidation, as well as a decrease in intracellular antioxidant enzymes. Provision of vitamin C and E simultaneously has been shown to reduce ROS and lipid peroxidation, which is supported by normal steroidogenic activity and antioxidant enzymes.

In a study conducted by Al-Olayan [14] regarding the effect of Pomegranate (*Punica granatum*) on testes of rats given carbon tetrachloride poisons, a significant increase in testosterone, antioxidant enzyme activity, and decreased lipid peroxidation after Pomegranate administration were found. The same results were also found in a study conducted by Chang [15] regarding the cytoprotective effect of *Morinda officinalis* on Leydig cells induced by hydrogen peroxide, showing an increase in antioxidant activity and an increase in testosterone production under oxidative stress conditions in Leydig cell culture in TM3 rats.

The decrease in testosterone levels in group 3 may also be caused by gamma-mangostin that can inhibit cell growth through the mechanism of intracellular ROS production and mitochondrial dysfunction as in the study conducted by Chang and Yang [16] in colorectal adenocarcinoma cells. Wang [17] mentioned in his study that gamma-mangostin with a level of 5 μg/mL could induce apoptosis and inhibit the G1 phase cell cycle in melanoma cells that were given behavior for 48 h. In another study, it was found that gamma-mangostin had an antiproliferative effect on human colon cancer cells DLD-1 at a level of 20 μM and incubated for 72 h through the S phase inhibition mechanism in the cell cycle [18].

In normal metabolism, Leydig cells produce ROS through an electron transport chain mechanism, and when steroid hydroxylation occurs by the cytochrome P450scc enzyme [13]. Jen [19] stated that ROS and activation of the mitochondrial apoptotic pathway could induce apoptotic initiator caspase-9, then caspase-9 would activate its effector, caspase-3. Kim [20] mentioned in his study that caspase-3 activation in Leydig cells led to Leydig cell apoptosis. Caspase-3 may play a role in the activation of core proteins that accelerate the final process of apoptosis, namely DNA fragmentation, which causes a gradual decrease in steroidogenesis activity by Leydig cells, as evidenced by the coloring of 3β-HSD [20].

The experiments conducted by Shakui et al. [16], in prostate cancer cells given hydroxanthone compounds extracted from the roots of the *Garcinia subelliptica* plant found an antiandrogenic effect on these cells. The chemical structure of the benzopyrene ring found in most xanthone compounds is capable of mediating the inhibitory process of the Sp-1 transcription factor found in the androgen receptor promoter (AR) and modifying posttranscriptional AR protein [21].

Another possibility that can cause no increase in testosterone levels in Leydig cell cultures is the low or lack of gamma-mangostin levels given. Nakatani [18] stated in his study that gamma-mangostin effectively inhibited the inflammatory process of C6 mouse glioma cells at a level of 10 μM. In this study, the gamma-mangostin levels used were 5 μM.

However, the small sample size in this study and only a single concentration of gamma-mangostin was given to the cell cultures are our main study limitations. Further study is necessary to investigate whether different concentrations of gamma-mangostin would decrease the toxic effect of AGE and increase testosterone levels. Finally, none of our findings showed that administration of gamma-mangostin could increase testosterone levels in Leydig cells culture of Sprague-Dawley rat induced by AGE.

## Conclusions

In conclusion, testosterone levels in Leydig cell cultures induced by AGE were lower than the control group. Giving gamma-mangostin 5 μM does not increase testosterone levels in Leydig cell cultures induced by AGE 200 μg/mL. Furthermore, this is the first study to examine the effect of gamma-mangostin administration on testosterone level of AGE-induced Leydig cell cultures. Further study with larger samples and different gamma-mangostin concentrations is important to confirm and clarify our findings.

## Data Availability

All data generated or analyzed during this study are included in the submission. The raw data are available from the corresponding author on reasonable request.

## Abbreviations

AGE: Advanced glycation end products
AR: Androgen receptor
BSA: Bovine serum albumin
DMSO: Dimethyl-sulfoxide
ELISA: Enzyme-linked immunosorbent assay
LH: Luteinizing hormone
PBS: Phosphate buffered saline
PCB: Polychlorinated biphenyl
ROS: Reactive oxygen species
SD: Standard deviation
WHO: World Health Organization
